# Changes in Mental Health and EEG Biomarkers of Undergraduates Under Different Patterns of Mindfulness

**DOI:** 10.1007/s10548-023-01026-y

**Published:** 2023-12-25

**Authors:** Miaoling Luo, Quan Gan, Ziyang Huang, Yunxiong Jiang, Kebin Li, Minxiang Wu, Dongxiao Yang, Heng Shao, Yanmei Chen, Yu Fu, Zhuangfei Chen

**Affiliations:** 1 Medical Faculty, Kunming University of Science and Technology, Kunming, China; 2 Brain Science and Visual Cognition Research Center, Medical School of Kunming University of Science and Technology, Kunming, China; 3 Faculté de Médecine, Université Paris-Saclay, Le Kremlin-Bicêtre, France; 4 Department of Geriatrics, The First People’s Hospital of Yunnan Province, Kunming, China

**Keywords:** Mindfulness, Mental Health, EEG biomarkers, Alpha, Low-beta

## Abstract

The effects of short-term mindfulness are associated with the different patterns (autonomic, audio guided, or experienced and certified mindfulness instructor guided mindfulness). However, robust evidence for reported the impacts of different patterns of mindfulness on mental health and EEG biomarkers of undergraduates is currently lacking. Therefore, we aimed to test the hypotheses that mindfulness training for undergraduates would improve mental health, and increase alpha power over frontal region and theta power over midline region at the single electrode level. We also describe the distinction among frequency bands patterns in different sites of frontal and midline regions. 70 participants were enrolled and assigned to either 5-day mindfulness or a waiting list group. Subjective questionnaires measured mental health and other psychological indicators, and brain activity was recorded during various EEG tasks before and after the intervention. The 5-day mindfulness training improved trait mindfulness, especially observing (p = 0.001, d = 0.96) and nonreactivity (p = 0.03, d = 0.56), sleep quality (p = 0.001, d = 0.91), and social support (p = 0.001, d = 0.95) while not in affect. Meanwhile, the expected increase in the alpha power of frontal sites (p < 0.017, d > 0.84) at the single electrode level was confirmed by the current data rather than the theta. Interestingly, the alteration of low-beta power over the single electrode of the midline (p < 0.05, d > 0.71) was difference between groups. Short-term mindfulness improves practitioners’ mental health, and the potentially electrophysiological biomarkers of mindfulness on neuron oscillations were alpha activity over frontal sites and low-beta activity over midline sites.

**Supplementary Information:**

The online version contains supplementary material available at 10.1007/s10548-023-01026-y.

## Introduction

During the pandemic, the mental health of college students was negatively impacted by anxiety, depressive symptoms, and acute stress, as shown by numerous studies (Bai et al. 2021; Huckins et al. 2020; Ma et al. 2020; Wu et al. 2021). Young people, particularly medical college students, were the primary victims of depression (Herrman et al. 2022; Thapar et al. 2022; Li et al. 2022). Moreover, college students inevitably encounter various stresses in studying during the COVID-19 outbreak, for the pace of life and learning habits were greatly affected. In view of the increasing demands on effective prevention and intervention strategies for the mental health of college students, through research, there is a great need to identify robust strategies for them.

Mindfulness involves nonjudgmentally attending to relevant aspects of experience, leading to a greater sense of emotional regulation, balance, and well-being (Ludwig & Kabat-Zinn 2008). Mindfulness training, including traditional forms such as mindfulness-based stress reduction (MBSR) and mindfulness-based cognitive therapy (MBCT), is becoming increasingly popular in universities (Creswell 2017). As both a skill and a therapy, mindfulness has been shown to improve symptoms of anxiety, depression, stress, and other mental states (Galante et al. 2018; Ji et al. 2021; Johannsen et al. 2022; Zhu et al. 2021). These therapies usually span 8 weeks. Brief mindfulness interventions might alter emotions and behaviours immediately following training (Creswell 2017). In addition, even a circa 15-min guided mindfulness instructions could change the brain activity (Andreu et al. 2018). Therefore, we focused on the effects of 5-day mindfulness training on trait mindfulness, positive or negative affect, sleep quality, as well as social support, and hypothesized that the 5-day mindfulness training showed positive improvements. The brain functional change reflects the above-mentioned effects can possibly be detected.

Over the past decade, there has been a growing body of literature demonstrating the impact of mindfulness on electroencephalography (EEG) results, for example, the alteration of brain waves pattern (Hauswald et al. 2015; Kang et al. 2022; Lomas et al. 2015; Saggar et al. 2012; Szumska et al. 2021), event-related potential (ERP) components (Atchley et al. 2016; Gupta et al. 2022; Sanger et al. 2018; Verdonk et al. 2020), functional connectivity (Berkovich-Ohana et al. 2014), brain network integration (van Lutterveld et al. 2017) and brain electric microstates (Zanesco et al. 2021). However, very few mindfulness practice combine with EEG studies were conducted in colleges students (An et al. 2022; A. S. Chan et al. 2008; Izhar et al. 2022). In this study, we highlight the patterns of EEG frequency bands as a potential neural oscillation marker in college students after 5-day mindfulness training and/or audio-guided mindfulness as an area primarily explored by early-stage studies.

A systematic review identified that most studies consistently reported that mindfulness is associated with increased alpha and theta power but not beta, delta and gamma power during or after mindfulness, especially in regular mindfulness practitioners (Lomas et al. 2015). In addition, the enhancement of alpha power is always correlated with lower anxiety, feelings of calm, positive affect and relaxation (Cahn & Polich 2006), and an increase of frontal midline theta (FMT) power is associated with internalized attention (A. S. Chan et al. 2008). Herein, it is reasonable to assert that the enhancement of frequency band power, mainly alpha power over the frontal region and theta power over the midline region, especially the frontal midline region, are directly correlated with mindfulness (Tang et al. 2019; Valadez 2015).

However, the previous studies adopted different brain regions to calculate the frequency bands’ power. These included whole brain regions (Kang et al. 2022; Lagopoulos et al. 2009), single site (S. H. W. Chan et al. 2021; Ng et al. 2021), and frontal, frontal midline, midline, temporal-central, posterior regions (Berkovich-Ohana et al. 2012; Hudak et al. 2021; Lagopoulos et al. 2009) and so on (Bigliassi et al. 2020; A. S. Chan et al. 2008). In the literatures reviewed above, there is variation in regional selection and methods used to calculate power spectrum. Four studies employed absolute power measurement. Among these, three reported mindfulness-related results primarily in the alpha and theta bands (including frontal midline theta, FMT) (Kang et al. 2022; S. H. W. Chan et al. 2021; Lagopoulos et al. 2009) and one reported result in the gamma band (Berkovich-Ohana et al. 2012). Another study repeated the results of alpha, theta, and FMT using power spectral density (PSD) combined with coherence analysis (Hudak et al. 2021). One study reported FMT results using relative power calculation (A. S. Chan et al. 2008). Two other studies reported changes in delta, beta, and low gamma power by using PSD (Ng et al. 2021) or coherence analysis (Bigliassi et al. 2020). Furthermore, among these studies, seven conducted EEG acquisition during mindfulness task, and one during a post-intervention cognitive task (Kang et al. 2022).

It has been suggested (A. S. Chan et al. 2008) that relative power measurements may produce larger estimates for the dominant frequency range (Klimesch, 1999) and that individual variations can be eliminated by computing the relative value for individual frequency band (A. S. Chan et al. 2007). However, it is important to consider the reproducibility of different methods for analyzing resting-state EEG measures (Duan et al. 2021). When deciding whether to choose indicators based on regions or connections, it is crucial to consider whether they are appropriate for the study's purpose, as our objective is to explore neural and behavioral representations (Horien et al. 2020) to gain a better understanding of how the brain shapes our experiences.

Therefore, we suppose that there are differences among each of the corresponding electrode of the frontal region and midline region, respectively, in alpha and theta frequency bands in power spectrum index and expect to find out which site has higher sensitivity and specificity among current EEG measures.

Previous studies have described the impact of mindfulness on psychological or EEG-related outcomes in students (Galante et al. 2018; Kuyken et al. 2013; Morais et al. 2021; Ren et al. 2011; Sanger et al. 2018). However, none of neuroscientific studies have explored whether and how brief mindfulness training influences certain psychological features especially social support in college students during the COVID-19 pandemic.

Therefore, we conducted the 5-day mindfulness training to assess whether provision of mindfulness courses to university students would improve their mental health states and describe the distinction among frequency bands patterns in different sites of frontal and midline regions in order to highlight whether alpha power over frontal region and theta power over midline region would increase after mindfulness training.

## Methods

### Participants

The study was approved by the Medical Ethics Committee, Kunming University of Science and Technology (KUST) (approval number: KMUST-MEC-142), and conducted this pilot non-randomized controlled trial in the Medical Faculty of KUST, China. Subjects were students attending the school who volunteered to join our study, and did not suffer pathologically or clinically diagnosed physical or mental disease (the total score of Patient Health Questionnaire-9 (S. Chen et al. 2013; Kroenke et al. 2001; Wang et al. 2014), PHQ-9 < 20; the total score of Generalized Anxiety Disorder-7 (Lin et al. 2021; Spitzer et al. 2006), GAD-7 < 15). We excluded students who generally have prior experience of mindfulness training. All participants were required to provide written informed consent and were given financial compensation for their participation. They were recruited from April 2021 to May 2021. From a total of 73 volunteers, a convenience sample consisting of 70 subjects met the inclusion criteria. The 36 students who completing baseline EEG recording in the first week were allocated to a free 5-day course of mindfulness training group (MTG). The detailed inclusions, exclusions, allocation of subjects is described in Supplementary file 2. All the participants were informed they would receive the same intervention without given a specific time. Fig. S1 (Supplementary file 3) presents the study flowchart.

### Group Training

The MTG was a face-to-face, group-base skills training programme consisting of five daily ~ 60-min sessions including one ~ 15-min exercises (sitting meditation, body scanning, mindfulness eating, mindful walking, loving-kindness meditation) that changed day to day and followed by experience sharing, as well as mindfulness meditation (e.g., in first day, a sitting meditation practice was induced with video guidance which lasts for about 15 min, then following a sharing of meditation experience for about 30 min. The face-to-face session was finally finish in mindful breathing meditation for about 5 min). The short-term mindfulness training mainly referred to regular 8-week MBSR practice and 4-week practice (Demarzo et al. 2017), as well as 5-day short-term meditation training (Tang et al. 2007). The waiting list group (WLG) were instructed to maintain their usual life activities but not engage with any mindfulness-related information until they had finished the study. Then they were informed they could receive intervention through discussing with the mindfulness instructor to confirm training start time in the next semester. Classes were taught by experienced instructor (JG) from Students Counseling and Mental Health Center at KUST.

### Questionnaires

The primary outcome was trait mindfulness, measured with the Five Factor Mindfulness Questionnaire (FFMQ) (Baer et al. 2006; Deng et al. 2011). The secondary outcomes included two parts. One was analyzed here, and the other one will be reported in other works. The Self-Rating Scale of Sleep (SRSS) (Li 2012), the Positive and Negative Affect Scale (PANAS) (Huang et al. 2003; Watson et al. 1988), and the Social Support Rate Scale (SSRS) (Xiao 1994) measured sleep quality, affect, and social support. Additional self-rated questionnaires will be reported concretely elsewhere, including a series of scales measuring other psychological indicators or behavioural habits and a self-designed questionnaire used to evaluate the state of the two groups during both baseline and post-intervention EEG recording or the mastery degree of the mindfulness-based skills after the intervention via participants’ self-rating. All the written questionnaires were collected at baseline and post-intervention measurement.

### EEG Acquisition

**Fig. 1:**
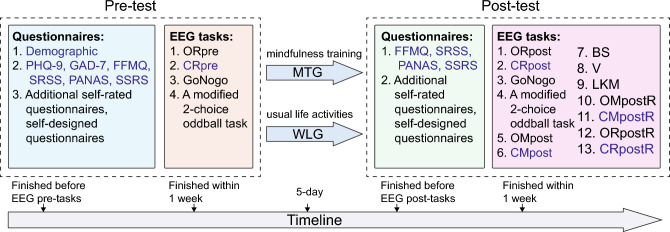
Experimental paradigms. MTG, mindfulness training group; WLG, waiting list group; PHQ-9: Patient Health Questionnaire-9; GAD-7: Generalized Anxiety Disorder-7; FFMQ: Five Factor Mindfulness Questionnaire; SRSS: Self-Rating Scale of Sleep; PANAS: Positive and Negative Affect Scale; SSRS: Social Support Rate Scale; the resting state with eyes open (OR) and eyes closed (CR); autonomic mindfulness practice with eyes open (OM) and eyes closed (CM); BS: body scanning; V: video; LKM: loving-kindness meditation. The content in blue font in the Questionnaires and EEG tasks box was reported in this paper, and the content in black font will be reported in other works

EEG was recorded during laboratory-based EEG paradigms programmed using E-prime software (Psychology Software Tools, Pittsburgh, PA). EEG data was recorded using the ActiveTwo BioSemi system (BioSemi, Amsterdam, The Netherlands). Recordings were taken from 16 scalp (Fp1, Fp2, F4, Fz, F3, T7, C3, Cz, C4, T8, P4, Pz, P3, O1, Oz, and O2) Ag/AgCl electrodes (10–20 International System) based on the 2-wire electrodes system, with the Common Mode Sense (CMS) active electrode and the Driven Right Leg (DRL) passive electrode, forming the ground electrode. The total bioelectric signals were digitized at a sampling rate of 2048 Hz and maintained below 40uV offsets from the CMS-DRL reference. A total of seventeen tasks were adopted for EEG recording, with four for baseline and thirteen for post-intervention. Four of the paradigms (i.e., go/nogo and oddball in both pre- and post-intervention measures) were not analysed in current report. Apart from to the eyes open (ORpre, ORpost) and eyes closed (CRpre, CRpost) EEG paradigms, which were administered with the same settings during both baseline and post-intervention measurements, a total of nine mindfulness tasks were assigned specifically for the post-intervention measurement to investigate the effects of the training. These tasks encompassed word instructed autonomic mindfulness task (OMpost, CMpost) (Hudak et al. 2021), body scanning, mindful self-video watching (participants watching a video of themselves when they are doing mindfulness training, with music playing in the background), loving-kindness meditation, additional mindfulness practice and additional resting state tasks (OMpostR, CMpostR, ORpostR, and CRpostR). (See Supplementary file 2 for details of EEG paradigms). Figure 1 presents the experimental paradigms.

### Data Analyses

#### Clinical Measures

All analyses were per the protocol and performed with SPSS (Version 20.0). The sample size of the final analysis was 63 (2 people withdrew from the study, 1 people did not receive the intervention, and two people’ post questionnaires were lost, all of them were in the MTG; 2 people lost to follow up in the WLG). Group differences in demographic variables were examined with unpaired *t*-tests or χ^2^ and Fisher Exact tests as appropriate. To determine differences in FFMQ, SRSS, PANAS and SSRS between groups, the data were inspected for two-sided unpaired *t*-tests, whereas within-group comparisons were made with two-sided paired *t*-tests.

#### EEG Preprocessing and Analyses

We employed EEGlab13.0.0b (https://sccn.ucsd.edu/eeglab) and custom MATLAB R2013b (The MathWorks, Natick, MA, United States) scripts to preprocess the EEG data offline. First, recorded EEG data were filtered at a range of 1–100 Hz followed by a notch filter at 50 Hz; then re-sampled to 512 Hz and epochs of 2 s were created for each subject. Contaminated epochs were discarded by visual inspection. Independent component analysis (ICA) was performed afterward.

After completing preprocessing, we excluded subjects who met the following criteria: contaminated ICs were more than 5 to be eliminated via the ADJUST plug-in (https://www.nitrc.org/projects/adjust/) or bad channel and bad segments with blinking times equal or greater than 10 within 1 min in all data length were removed continuously using visual inspection, which was considered as continuous eye-opening state due to participants not follow the instructions. And then, the processed data (MTG = 18 and WLG = 15) was converted into frequency domain representations using the Darbeliai plug-in (v2019.02.01.1; https://github.com/embar-/eeglab_darbeliai/wiki/0.%20EN) in EEGlab (https://sccn.ucsd.edu/eeglab) to calculated average EEG band powers, including Abs, Rel, and PSD (See Supplementary file 2). Log10 transformation is performed when necessary to reduce heteroscedasticity.

To test differences in log_10_-transformed Abs (Abs/lg), Rel, and PSD in all kinds of EEG bands (delta: 1–4 Hz, theta: 4–8 Hz, alpha: 8–13 Hz, low-beta: 13–20 Hz, high-beta: 20–30 Hz, and low-gamma: 30–48 Hz) in the frontal (Fp1, Fp2, F3, Fz, and F4) and midline (Fz, Pz, and Oz) regions, where Fz is the frontal midline electrode, of five EEG tasks (CRpre, CRpost, CMpost, CMpostR, and CRpostR) between groups, respectively, data were inspected for two-sided unpaired *t*-tests, while within-group comparisons were made with multiple paired *t*-test using Bonferroni’s adjustment. For each *t*-test either in clinical measures or EEG analysis, the effect size was calculated (i.e., unbiased cohen’s d).

## Results

### Demographic and Clinical Characteristics

Sixty-three participants (42 females) were analysed in this study, of whom 31 (20 females and 11 males, aged 19.3 ± 0.7 years) served as the MTG and 32 (22 females and 10 males, aged 19.3 ± 0.8 years) served as the WLG (Table 1). No significant difference was found comparing the baseline demographic and screening scales (PHQ-9 and GAD-7) before mindfulness training between the two groups (Table 1).

**Table 1:** Baseline demographic and clinical characteristics (N = 63) of undergraduates in two groups

Characteristic	MTG	WLG	Test statistic	P-value
N (%)	31 (49.2%)	32 (50.8%)	NA	NA
Sex, N (%)			χ^2^ = 0.13	0.72
Female	20 (64.5%)	22 (68.8%)		
Male	11 (35.5%)	10 (31.2%)		
Age, M ± SD	19.3 ± 0.7	19.3 ± 0.8	t = − 0.28	0.78
Nation, N (%)			χ^2^ = 0.43	0.51
Han nationality	22 (71.0%)	25 (78.1%)		
National minority	9 (29.0%)	7 (21.9%)		
Handedness, N (%)			χ^2^ = 2.09	0.51
Right handedness	28 (90.3%)	30 (93.8%)		
Left handedness	2 (6.5%)	0 (0.0%)		
Other	1 (3.2%)	2 (6.2%)		
Major, N (%)			χ^2^ = 2.79	0.10
Clinical medicine	29 (93.5%)	24 (75.0%)		
Nursing	2 (6.5%)	8 (25.0%)		
Grade, N (%)			χ^2^ = 0.76	0.38
Freshman	26 (83.9%)	24(75.0%)		
Sophomore	5 (16.1%)	8 (25.0%)		
BMI, M ± SD	20.0 ± 2.5	20.8 ± 3.1	t = − 1.03	0.31
Smoking, N (%)			χ^2^ = 1.00	0.68
Yes	2 (6.5%)	2 (6.2%)		
No	29 (93.5%)	30 (93.8%)		
Alcohol use, N (%)			χ^2^ = 0.67	0.35
Yes	2 (6.5%)	4 (12.5%)		
No	29 (93.5%)	28 (87.5%)		
Score, M ± SD				
PHQ-9	6.4 ± 3.7	7.4 ± 3.7	t = − 1.02	0.31
GAD-7	5.9 ± 3.5	6.0 ± 3.1	t = − 0.19	0.85

*MTG* mindfulness training group; *WLG* waiting list group; *NA* not applicable; *M* mean; *SD* standard deviation; *BMI* body mass index; *PHQ*-9 Patient Health Questionnaire-9; *GAD*-7 Generalized Anxiety Disorder-7

MTG improved observing_post_ (p = 0.002, d = 0.81), observing_Δpost-pre_ (p = 0.000, d = 1.02), SSRS total_post_ (p = 0.02, d = 0.67), and objective support_post_ (p = 0.02, d = 0.67) after mindfulness training compared with WLG applying unpaired two-tailed t-tests, while there were no significant differences between groups in clinical characteristics in any other measures assessed (Table 2). Within-group analysis showed that after the mindfulness training intervention, the MTG exhibited a significant increase in the FFMQ total score (p = 0.03, d = 0.56), observing score (p = 0.001, d = 0.96), nonreactivity score (p = 0.03, d = 0.56), SSRS total score (p = 0.001, d = 0.95), and subjective support score (p = 0.03, d = 0.57) and a decrease in the SRSS total score (p = 0.001, d = 0.91) from the scores before the intervention, whereas all the scales scores of the WLG remained unchanged (Table 2). For each group, no significant difference was observed in the PANAS score (Table 2).

**Table 2:** Questionnaires statistics (Mean ± SD)

Measure	MTG (n = 31)^a^	WLG (n = 32)^a^	Statistics	P-value^b^
FFMQ				
Total_pre_	115.35 ± 10.858	115.69 ± 11.206		0.91
Total_post_	119.45 ± 12.441 t = 2.26; p = 0.03^*^ **(d = 0.56)**	114.94 ± 9.922 t = − 0.40; p = 0.69 (d = 0.10)		0.12
Total_Δpost-pre_	4.10 ± 10.104	− 0.75 ± 10.635	t = 1.85	0.07
Observing_pre_	24.90 ± 4.650	25.03 ± 5.301		0.92
Observing_post_	28.00 ± 5.132 t = 3.80; p = 0.001^**^ **(d = 0.96)**	23.84 ± 5.030 t = − 1.79; p = 0.08		0.002^**^(d = 0.81)
Observing_Δpost-pre_	3.10 ± 4.541	− 1.19 ± 3.763	t = 4.08	0.001^***^(d = 0.98)
Describing_pre_	22.74 ± 4.058	22.78 ± 5.393		0.97
Describing_post_	23.65 ± 5.148 t = 1.29; p = 0.21	23.25 ± 4.392 t = 0.68; p = 0.50		0.74
Describing_Δpost-pre_	0.90 ± 3.910	0.47 ± 3.927	t = 0.44	0.66
Nonjudging_pre_	21.45 ± 4.508	21.66 ± 4.830		0.86
Nonjudging_post_	20.52 ± 5.507 t = − 1.33; p = 0.19	21.56 ± 5.769 t = − 0.12; p = 0.91		0.47
Nonjudging_Δpost-pre_	− 0.94 ± 3.915	− 0.09 ± 4.431	t = − 0.80	0.43
Nonreactivity_pre_	20.06 ± 3.768	19.47 ± 3.233		0.50
Nonreactivity_post_	21.13 ± 4.006 t = 2.24; p = 0.03^*^ **(d = 0.56)**	20.28 ± 3.401 t = 1.60; p = 0.12		0.37
Nonreactivity_Δpost-pre_	1.06 ± 2.645	0.81 ± 2.867	t = 0.36	0.72
Acting with awareness_pre_	26.19 ± 5.056	26.75 ± 4.340		0.64
Acting with awareness_post_	26.16 ± 4.845 t = − 0.04; p = 0.97	26.0 ± 4.048 t = − 0.84; p = 0.41		0.89
Acting with awareness_Δpost-pre_	− 0.03 ± 4.119	− 0.75 ± 5.074	t = 0.62	0.54
PANAS				
Negative_pre_	21.19 ± 5.600	22.31 ± 6.503		0.47
Negative_post_	21.45 ± 6.355 t = − 0.25; p = 0.80	22.38 ± 6.852 t = − 0.06; p = 0.95		0.58
Negative_Δpre-post_	− 0.26 ± 5.744	− 0.06 ± 6.080	t = − 1.31	0.90
Positvie_pre_	28.58 ± 4.478	27.28 ± 6.139		0.34
Positvie_post_	29.90 ± 5.700 t = 1.88; p = 0.07	27.59 ± 7.089 t = 0.26; p = 0.79		0.16
Positvie_Δpost-pre_	1.32 ± 3.919	0.31 ± 6.713	t = 0.73	0.47
SRSS				
SRSS_pre_	21.74 ± 4.008	21.59 ± 4.613		0.89
SRSS_post_	19.58 ± 3.931 t = − 3.61; p = 0.001^**^ **(d = 0.91)**	20.66 ± 4.555 t = 1.43; p = 0.16		0.32
SRSS_Δpre-post_	2.16 ± 3.338	0.94 ± 3.706	t = 1.38	0.17
SSRS				
Total_pre_	38.39 ± 4.364	36.13 ± 4.549		0.05
Total_post_	39.68 ± 5.294 t = − 3.78; p = 0.001^**^ **(d = 0.95)**	36.44 ± 4.996 t = − 0.40; p = 0.69		0.02^*^(d = 0.67)
Total_Δpost-pre_	1.29 ± 1.901	0.31 ± 4.373	t = 1.16	0.25
Objective support_pre_	8.81 ± 1.887	8.13 ± 1.737		0.14
Objective support_post_	9.32 ± 2.072 t = − 1.89; p = 0.07	8.22 ± 1.408 t = − 0.29; p = 0.78		0.02^*^(d = 0.67)
Objective support_Δpost-pre_	0.52 ± 1.525	0.09 ± 1.855	t = 0.99	0.33
Subjective support_pre_	21.16 ± 2.806	20.03 ± 3.412		0.16
Subjective support_post_	21.77 ± 3.127 t = − 2.28; p = 0.03^*^ **(d = 0.57)**	20.44 ± 3.232 t = − 0.75; p = 0.46		0.10
Subjective support_Δpost-pre_	0.61 ± 1.498	0.41 ± 3.078	t = 0.34	0.74
The rate of social support utilization_pre_	8.42 ± 1.544	7.97 ± 1.448		0.24
The rate of social support utilization_post_	8.58 ± 1.840 t = − 0.62; p = 0.54	7.78 ± 1.827 t = − 0.66; p = 0.52		0.09
The rate of social support utilization_Δpost-pre_	0.16 ± 1.440	− 0.19 ± 1.615	t = 0.90	0.37

*SD* standard deviation; *MTG* mindfulness training group; *WLG* waiting list group; *FFMQ* Five Factor Mindfulness Questionnaire; *PANAS* Positive and Negative Affect Scale; *SRSS* Self-Rating Scale of Sleep; *SSRS* Social Support Rate Scale

^a^p-values from two-tailed paired t-test

^b^p-values from two-tailed unpaired t-test

*p < 0.05; **p < 0.01; ***p < 0.001; for each t-test, the effect size (d) was caculated (i.e. unbiased cohen’s d)

### EEG Power and Spectrum

**Fig. 2:**
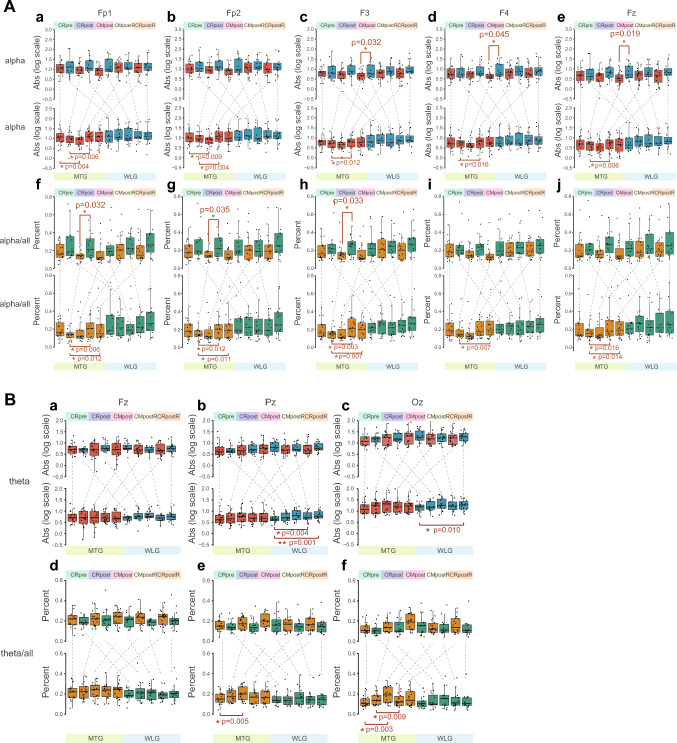

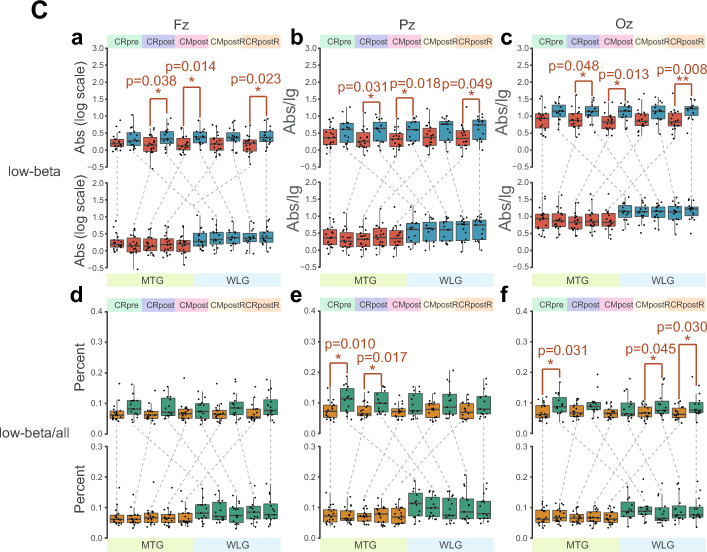
Box-plots of absolute and relative powers of alpha (**A**), theta (**B**) and low-beta (**C**) over frontal or midline regions on 5 EEG tasks between and within groups. MTG, mindfulness training group; WLG, waiting list group; the resting state with eyes closed (CR); autonomic mindfulness practice with eyes closed (CM); log scale, means log10-transformed; the upper portion represent absolute power (Abs) while the lower represent relative power at frontal or midline regions; the two rows on powers’ box-plots represent results between groups analysis (two-tailed unpaired t-test, *p < 0.05; **p < 0.01; ***p < 0.001)and within group analysis (multiple paired t-test with Bonferroni correction based on CRpre: *p < 1.25 × 10^–2^, **p < 2.5 × 10^–3^, ***p < 2.5 × 10^–4^; and CRpost: *p < 1.67 × 10^–2^, **p < 3.3 × 10^–3^, ***p < 3 × 10^–4^). For each t-test, the effect size was calculated (i.e., unbiased cohen’s d)

The effect of mindfulness training on eyes-closed resting-state EEG activity and the effect of ongoing autonomic mindfulness training (i.e., adopted mindfulness skill, not limited to instruction) on eyes-closed EEG activity were examined. The results of Abs/lg, Rel, and PSD were showed in Fig. S2 and S3 (Supplementary file 4, 5), Fig. S4, S5 and S6 (Supplementary file 6–8), and Fig. S7 (Supplementary file 9), separately. Their description was exhibited in Supplementary file 1. Due to our hypothesis and the find of interest in post hoc analysis, we primarily reported the results of alpha power over frontal region, theta and low-beta powers over midline region. Namely, within-group analysis showed that the alpha Abs/lg in the MTG group increased significantly during autonomic mindfulness task in post-intervention stage (CMpostR) at all sites after the day’s audio and video guidance (p < 0.017) (Fig. 2A). While the theta power over midline region presented no regular change both in between-group or within-group analysis (Fig. 2B). Interestingly, low-beta Abs/lg power of MTG was significantly lower than WLG over midline region (p < 0.05) (Fig. 2C). (Details for the above results were presented in supplementary file 2.)

## Discussion

This study examined the efficacy of a 5-day mindfulness training course for the improvement of mental health and the alteration of brain wave activity in a healthy group of Chinese university students. The results supported this course as an effective intervention for improving trait mindfulness, social support, sleep quality, and increasing alpha power of frontal sites at the single electrode level among all EEG measures during the autonomic mindfulness task (CMpostR) following audio and video instruction, but not theta. In addition, there was a significant difference between two groups in low beta Abs/PSD over midline regions during post-intervention tasks except CMpostR. However, no one location in the above bands appeared to have higher sensitivity and specificity among these three types of EEG measures.

### Mental Health-Related Outcomes

Our results suggest that the 5-day mindfulness training course improved trait mindfulness—our primary outcome, particularly observing and nonreactivity. It was in line with another study, which found that an 8-week web-based mindfulness could increase mindfulness scores (El Morr et al. 2020). In contrast, others found that a 6-week mindfulness intervention was not associated with improved trait mindfulness in medical students (Damião Neto et al. 2020). One reason for the different effects might be the mindfulness experience of conductors and participants.

Our analysis also showed an increase in SSRS total score, objective and subjective support scores, and a decrease in SRSS total score in MTG while it was absence of significant difference in the PANAS score. The finding about SSRS is among the first to examine the effects of 5-day mindfulness training in social support which may be related to human well-being (Chen et al. 2021). In terms of sleep quality, the reduction in total SRSS score in MTG was consistent with the findings of a systematic review and meta-analysis of randomized controlled trials (RCTs), which showed that mindfulness meditation interventions significantly improved sleep quality (Rusch et al. 2019). For PANAS score, previous trials indicate that mindfulness intervention might improve positive affect (Bossi et al. 2022) and negative affect (Glück & Maercker 2011). In contrast, the present study showed no difference in affect, which might be due to the short period of mindfulness training, or the small sample size. The positive affect score (p = 0.07) tended to be raised after intervention in MTG (Table 2), although this was not significant. Chilver and colleagues found that high alpha and delta power combined with low beta power was associated with elevated wellbeing (Chilver et al. 2020). This means that there is likely a more subtle relationship between mental health, wellness and brain activity. In the context of neurofunction, mental health outcomes should be further analysed. Our experiment was conducted during the lockdown period of the epidemic, with everyone maintaining a certain degree of social distancing even while on campus. During the 5-day mindfulness training, social relationships were reestablished from a state of social distancing, which may also be a confounding factor that cannot be ignored. However, the mindfulness training we are exploring is in the form of group training, which is a fundamental element of the 5-day short-term mindfulness. Therefore, it is undeniable that in the context of social distancing, the reasonable provision of mindfulness group training does indeed benefit students. However, as observed during the 5-day training, the complexity and randomness cannot be ruled out.

### Neural Oscillation-Related Outcomes

The discrepancies in EEG frequency bands Abs, Rel, and PSD were showed between two groups (Fig. S2, S4, and S7b) (Supplementary file 4, 6, 9) and between the post-test and pre-test in each group (Fig. S3, S5, and S7a) (Supplementary file 5, 7, 9), and we could easily see that the results of Abs and PSD were almost the same. Of the hypothesized electrophysiological effects of the mindfulness training, which expected increase in the alpha of frontal sites (Fig. 2A) at the single electrode level was confirmed by the current data rather than the theta (Fig. 2B). It is worth to be mentioned that during the mindfulness (CMpostR) and resting (CRpostR) tasks, compared with resting (CRpost) on the same day, subjects who had participated in the mindfulness training showed significantly increased in alpha Abs, Rel, PSD and Rel at almost all sites, respectively, whereas similar gains were not found for the waiting list group, which is consistent with the results of most literature (Bing-Canar et al. 2016; Cahn and Polich 2006; Lomas et al. 2015; Tarrant et al. 2018). This suggests that participants in MTG were likely to be more relaxed than in WLG when under the guidance of the audio mindfulness materials, or shortly after. For theta power, there was no significant difference between the two groups, most likely because only experienced mindfulness has this marker, which is different from mindfulness novices and non-mindfulness practitioners (Lomas et al. 2015). In contrast, MTG and WLG both had significant differences between two EEG-related tasks of Pz or Oz, which could be due to some subjects feeling tired when performing time-consuming tasks (van den Berg et al. 2005).

It is interesting to note that the second apparent indicator of significance for between-group analysis in Abs and PSD was low-beta over the single midline electrode, whereas for within-group analysis there was little significant difference (Fig. 2C, S7). The two groups were not consistent in low-beta Rel at CRpre, which may be due to some ambiguities in the quantification of relative power (Chilver et al. 2020). Nevertheless, these results indicate an emergent feature of brain activity after 5 days of mindfulness training (Cahn and Polich 2006). It would possibly be a result of a generate state of brain activity and/or rapid repetition itself (Cahn and Polich 2006; Cook 1946), which happens in WLG, i.e., the low-beta oscillation could be induced by audio guidance and/or during CMpostR. Nevertheless, mindfulness may not entirely contribute to the pattern. The post-intervention assessments of MTG would be a “superposition state” of immediate effects of mindfulness and the task paradigm, which is need to decompose in order to distinguish the accurate effect of mindfulness. Overall, low-beta power may become a trait marker of brain activity to distinguish mindfulness practitioners after mindfulness training, with a limited time threshold from non-mindfulness practitioners, or mindfulness novices, who have not reached enough mindfulness training (Ng et al. 2021). The effect would be sensitively detected in single task status. We feel this is worthy of systematic and in-depth exploration in the future.

For delta, high-beta and low-gamma power bands in some studies, show some inconsistency, with reports of increases and decreases and no differences (Cahn & Polich 2006; Lomas et al. 2015). Likewise, with these bandwidths-related results in current study (Fig. S2–7) (Supplementary file 4–9), we found it hard to find obvious patterns or specificity related to the individual differences (For example, differences may relate to the sleepiness and fatigue of the subjects. After all, the post-test EEG took a long time (about 2 h), and some of the subjects reported feeling tired and falling asleep for a short time), or some random phenomena arising from the sample size reduction. This suggests that these bands may not represent the functions of mindfulness.

As to different measures of neural oscillation, it should be carefully considered when explanation. (1) The comparison between the alpha absolute and relative power groups revealed that the MTG had lower alpha power than the WLG (Supplementary file 1, 2), indicating higher levels of relaxation in WLG. These findings may attribute to the fact that mindfulness exercises not only induce a relaxation response but also require the allocation of cognitive resources to maintain focus, particularly for beginners. Additionally, the WLG group did not require an understanding of the mindfulness exercise content, which may have contributed to their higher relaxation levels. However, further analysis is required to explore subjective reports of alertness and relaxation levels. (2) The disparity between absolute power and relative power in the frontal lobe single electrode region may be attributed to the calculation method of absolute power, which is not normalized and can be influenced by significant individual variations, thus potentially affecting its repeatability. As such, to enhance the comparability of research findings, it is recommended that future studies focus primarily on relative power (Duan et al. 2021).

### Limitations and Future Research

The limitations of the current study are the relatively small sample size and only 16 EEG electrodes, limiting the EEG data quality screening process. The smaller sample size for analysis, might not rule out the phenomenon of statistical false positives or false negatives. Second, the low-gamma results in this paper might lack reliability due to the limitation of the hardware equipment. In addition, the cognition-related frequencies are mainly below 30 Hz (Chikhi et al. 2022), and EEG beyond these frequency bands is easily confused with the electromyogram (EMG) (Sleigh et al. 2001). Third, the analysis of EEG oscillations did not explore the relationships with brain structure as well as source analysis (Lantz et al. 2003). Fourth, there are different training period in the mindfulness field, diversified training paradigms, and many kinds of mindfulness techniques. It cannot guarantee that our own mindfulness training courses maintain a high degree of consistency with other researchers, which makes the repeated verification of the research challenging. This needs to be standardized in the future. Finally, our data analysis showed the power of each frequency band in a single channel, which makes for a lack of strong contrast with the results of average whole brain region, or average brain region of interest for most researchers. This study is relatively simple and may miss other valuable specific indicators compared to the other research of extended analysis on the ratio of various frequency bands (Shanok et al. 2022). Further complex indicators need to be accessed in future analysis, e.g., direct evidence of internalized attention which related to EEG alterations should be probed.

Future research could explore various EEG-related analysis indicators or synchronously combine functional magnetic resonance imaging (fMRI), functional near-infrared spectroscopy (fNIRS), magnetoencephalography (MEG), etc. to form multimodality analysis that could compensate the weakness of each detecting methods, and further explore the relationship between EEG indicators and subjective measurement indicators, to find more specificity.

### Supplementary Information

Below is the link to the electronic supplementary material.

Supplementary file1 (DOCX 21 KB)—The description of the distinction among frequency bands patterns in different sites of frontal and midline regions.

Supplementary file2 (DOCX 19 KB)—Supplementary detailed information on methods and results.1) The inclusions and exclusions of subjects; 2) Instruction for post-intervention EEG paradigms; 3) EEG pre-processing and the calculation of the three EEG measures of brain activities; 4) The Results of EEG Power and Spectrum.

Supplementary file3 (PDF 864 KB)—Study flowchart. MTG, mindfulness training group; WLG, waiting list group; ICs: independent components.

Supplementary file4 (PDF 1658 KB)—The heatmap and box-plots of log10-transformed absolute power for between-group analysis results. (a) Heatmap of absolute power of five frequency bands in sites of frontal and midline regions. Abs/lg bar: values are depicted as standardized Z-scores for each power, where blue represents low value and purple represents high value. (b) Box-plots of absolute power between groups (two-sided unpaired t-tests; *p < 0.05, **p < 0.01, ***p < 0.001). Note: MTG, mindfulness training group; WLG, waiting list group; the resting state with eyes closed (CR); autonomic mindfulness practice with eyes closed (CM); five frequency bands: delta, theta, alpha, low-beta, high-beta, and low-gamma bands; frontal region: Fp1, Fp2, F3, Fz, and F4; midline region: Fz, Pz, and Oz; Fz is the frontal midline electrode; EEG tasks: CRpre, CRpost, CMpost, CMpostR, and CRpostR. Abs/lg, means log10-transformed absolute power.

Supplementary file5 (PDF 1242 KB)—The box-plots of log10-transformed absolute power for within-group analysis results. Data were inspected for multiple paired t-test (four pairwise comparisons based on CRpre: *p < 1.25×10^-2^, **p < 2.5×10^-3^, ***p < 2.5×10^-4^; three pairwise comparisons based on CRpost: *p < 1.67×10^-2^, **p < 3.3×10^-3^, ***p < 3×10^-4^, both following Bonferroni correction). See Figure S2 Note for the acronym.

Supplementary file6 (PDF 1454 KB)—The boxplots of relative power of five frequency bands in sites of frontal and midline regions between groups.(a) Heatmap of relative power of five frequency bands in sites of frontal and midline regions. Rel bar: values are depicted as standardized Z-scores for each power, where blue represents low value and purple represents high value. (b) Box-plots of relative power between groups (two-sided unpaired t-tests; *p < 0.05, **p < 0.01, ***p < 0.001). Rel, relative power; delta/all, theta/all, alpha/all, low-beta/all, high-beta/all, and low-gamma/all, representing delta, theta, alpha, low-beta, high-beta, and low-gamma bands’ relative power, respectively; see Figure S2 Note for the acronym.

Supplementary file7 (PDF 1180 KB)—The box-plots of the relative power for within-group analysis results.Data were inspected for multiple paired t-test (four pairwise comparisons based on CRpre: *p < 1.25×10^-2^, **p < 2.5×10^-3^, ***p < 2.5×10^-4^; three pairwise comparisons based on CRpost: *p < 1.67×10^-2^, **p < 3.3×10^-3^, ***p < 3×10^-4^, both following Bonferroni correction). See Figure S2 Note for the acronym.

Supplementary file8 (PDF 2330 KB)—Topographic maps showing scalp recorded relative power of five frequency bands for five EEG tasks under the eyes closed state. Scales: [0.15-0.51], [0.14-0.46], [0.05-0.10], [0.06-0.14], [0.02-0.11], [0.02-0.24]. See Figure S2 Note for the acronym.

Supplementary file9 (PDF 1236 KB)—The line charts of the power spectral density for between- and within-group analysis results. (a) The PSD of five frequency bands in sites of frontal and midline regions within groups. Data were inspected for multiple paired t-test (pairwise comparisons based on CRpre: *p < 1.25×10^-2^, **p < 2.5×10^-3^, ***p < 2.5×10^-4^; and CRpost: *p < 1.67×10^-2^, **p < 3.3×10^-3^, ***p < 3×10^-4^, both following Bonferroni correction). (b) The PSD between groups (two-sided unpaired t-tests; *p < 0.05, **p < 0.01, ***p < 0.001). frequency bands: 1-4Hz, 4-8Hz, 8-13Hz, 13-20Hz, 20-30Hz, and 30-48Hz, representing delta, theta, alpha, low-beta, high-beta, and low-gamma bands, respectively; See Figure S2 Note for the acronym.

## Data Availability

The data generated for this study are available from the corresponding author upon reasonable request.
