# Supplementary material for: Changes in Mental Health and EEG Biomarkers of Undergraduates Under Different Patterns of Mindfulness
Source: Brain Topogr. 2023 Dec 25;37(1):75–87. doi: 10.1007/s10548-023-01026-y (PMC10771601; doi:10.1007/s10548-023-01026-y)
Supplement: Supplementary file 1 — Supplementary file1 (DOCX 21 KB)—The description of the distinction among frequency bands patterns in different sites of frontal and midline regions. [file 10548_2023_1026_MOESM1_ESM.docx]

**The description of the distinction among frequency bands patterns in different sites of frontal and midline regions**

Our analysis below will consist of a pre- and post-intervention comparison between two groups (pre-MTG vs pre-WLG and post-MTG vs post-WLG) and a pre- and post-intervention comparison within two groups (pre-MTG vs post-MTG and pre-WLG vs post-WLG) according to three categories of EEG data: absolute power, relative power and power spectral density.

**For log_10_-transformed absolute power (Abs/lg)**

- Between-group analysis results (see Fig. S2) shows that in alpha and low-beta bands had significant differences, details in Fig. S2. Within alpha band, both groups showed significant differences in CMpost task, compare to WLG the electrode of Fz, F3, F4 from MTG had significant weakened signal. Within low-beta band, both groups showed significant differences in CRpost, CMpost and CRpostR tasks, which also showed the same trend as alpha band, the electrode of Fz, Pz, Oz from MTG had significant weakened signal. The other electrode absolute power had no significant differences in 5 serial tasks (in comparison between MTG and WLG). For high-beta and low-gamma band there was only in the electrode of Oz in CRpostR task showed significant difference.
- Within-group analysis results (see Fig. S3) shows that after intervention,

(1). The Abs/lg of alpha band from the MTG after the audio-video guided mindfulness of the day increased significantly, and The Abs/lg of alpha band of all channels increased on CMpostR compared to CRpost. in addition to this between different task we also observed some variation in electrode of Pz showed higher absolute power in CRpostR than in CRpost, in electrode of Fp1 and Fp2 showed higher absolute power in CRpre than in CMpost, and obviously there was no difference within WLG;

(2). The Abs/lg of theta band within WLG in electrode of Pz showed CRpostR and CMpostR was raised up compare to CRpre, in electrode of Oz showed CRpostR was raised up compare to CRpre. The rest other electrodes showed no significant difference within MTG.

(3). The Abs/lg of delta band within MTG in electrode of Fz and Pz showed CRpostR was dropped down compare to CRpre, in electrode of F3 showed CMpostR was dropped down compare to CRpre. Within WLG in the task of CRpostR and CMpostR of electrode Pz and Oz had raised up compare to CRpre.

(4). There was no significant difference within both MTG and WLG on low-beta band of all sites.

(5). There was also no significant difference within both MTG and WLG on high-beta band and low-gamma bands of all sites except Fp1 and Oz, which exhibited that in high-beta the Abs/lg of CRpostR was decreased than CRpost in WLG while no significant difference was found within the MTG and in low-gamma the Abs/lg of CRpostR was decreased than CRpost in WLG whereas the Abs/lg of CMpost and CRpostR was both decreased than CRpost within MTG.

Fig. S2. The heatmap and box-plots of log_10_-transformed absolute power for between-group analysis results

Fig. S3. The box-plots of log_10_-transformed absolute power for within-group analysis results

**For relative power**

- Between-group analysis (see Fig. S4) showed that the average activity for the low-gamma/all ratio was significantly different between the two groups over all sites in certain kinds of tasks. There was no significant difference between MTG and WLG bands on the low-beta power values of all sites except Pz and Oz. There was also no significant difference between the two groups in the delta bands power value of all sites except Fp1 and F3. Noticeably, there was a significant difference between MTG and WLG on the relative alpha band power value over Fp1, Fp2, and F3 but not over other sites. No significant difference was observed between MTG and WLG on the relative theta and high-beta bands power value of all sites.
- Within-group analysis (see Fig. S5) showed that

(1). The MTG after mindfulness intervention had a significant increase in the relative alpha band power value in the frontal area after the audio-video guided mindfulness on the day, which showed that except for the Pz and Oz electrodes, the relative alpha band power value on the other electrodes were increased on CRpostR compared to CRpost, and an increase of Fz, Fp1, Fp2, F3 were also detected between CMpostR and CRpost;

(2). Relative delta band power value decreased on Fz of WLG as CRpostR vs CRpre but no significant difference between other channels and tasks; on Fz of MTG as CRpostR vs CRpre, CRpostR vs CRpost on Fp2, CRpostR and CMpostR on F3 vs both CRpost and CMpostR vs CRpre, and CRpostR on F4 vs CRpost, while there was no significant difference between the individual tasks on Pz, Oz and Fp1;

(3). There was no significant difference in both MTG and WLG on relative theta, high-beta, and low-gamma bands power value of all sites except Pz or/and Oz, which exhibited that in Pz the relative theta band power value of CMpost was increased than CRpre in MTG and the relatively low-gamma band power value of CRpostR was decreased than CRpost in MTG, and in Oz the relative theta band power value of CMpost was increased than CRpost in MTG. Besides, CMpost was also increased than CRpre. The relatively high-beta and low-gamma bands power value of Oz in CRpostR of MTG were both decreased than CRpost. Besides, the relative low-gamma band power value of Oz in CMpostR was also decreased than CRpost. There was no significant difference in both MTG and WLG on the relatively low-beta band power value of all sites.

Fig. S4. The heatmap and box-plots of the relative power for between-group analysis results

Fig. S5. The box-plots of the relative power for within-group analysis results

Fig. S6. Topographic maps showing scalp recorded relative power of all frequency bands for five EEG tasks under the eyes closed state.

**For power spectral density (PSD)**

- Between-group analysis (see Fig. S7a) showed that the differences between MTG and WLG in all five tasks of all sites revealed the following:

(1) In CRpre, low-beta band PSD was significantly different between two groups at Oz;

(2) In CRpost, low-beta band PSD was significantly different between two groups at both Fz and Pz, while alpha band PSD was significantly different between two groups only at Fz;

(3) In CMpost, low-beta band PSD was significantly different between two groups at Fz, Pz, and Oz whereas alpha band PSD was significantly different between two groups at Fp1, F3, F4, and Fz;

(4) In CMpostR, no significant difference was observed between MTG and WLG on all bands PSD of all sites;

(5) In CRpostR, low-beta band PSD was significantly different between two groups at Fz (p = 0.028) and Oz (p = 0.010) while high-beta and low-gamma bands PSD were both significantly different between two groups only at Oz.

- Within-group analysis (see Fig. S7b) showed that the differences in each band PSD of five tasks of all sites in both MTG and WLG revealed the following:

(1) For MTG, the delta band PSD was only significantly decreased at Fz whereas alpha band PSD was significantly different between certain kinds of tasks of all sites. And low-gamma band PSD was significantly decreased at Oz and Fp1;

(2) For WLG, there was significantly difference at Pz, Oz, Fp1, and F4 while no significant difference was observed in six bands PSD in all five tasks of Fz, Fp2, and F3.

Fig. S7. The line charts of the power spectral density for between- and within-group analysis results
