# Supplementary material for: Changes in Mental Health and EEG Biomarkers of Undergraduates Under Different Patterns of Mindfulness
Source: Brain Topogr. 2023 Dec 25;37(1):75–87. doi: 10.1007/s10548-023-01026-y (PMC10771601; doi:10.1007/s10548-023-01026-y)
Supplement: Supplementary file 2 — Supplementary file2 (DOCX 19 KB)—Supplementary detailed information on methods and results.1) The inclusions and exclusions of subjects; 2) Instruction for post-intervention EEG paradigms; 3) EEG pre-processing and the calculation of the three EEG measures of brain activities; 4) The Results of EEG Power and Spectrum. [file 10548_2023_1026_MOESM2_ESM.docx]

**The inclusions and exclusions of subjects**

We included current undergraduate students (age≥18 years) with normal vision or corrected to normal at the Medical Faculty of KUST, and students who did not suffer pathologically or clinically diagnosed physical or mental disease (the total score of Patient Health Questionnaire-9 (S. Chen et al., 2013; Kroenke et al., 2001; Wang et al., 2014), PHQ-9<20; the total score of Generalized Anxiety Disorder-7 (Lin et al., 2021; Spitzer et al., 2006), GAD-7<15). We excluded students who generally have prior experience of mindfulness training; are a tobacco or any substance abuse; not suitable for electroencephalography (e.g., metallic implants, severe extracranial injuries or electrode allergy); the total score of PHQ-9≥20; the total score of GAD-7≥15; adherent to a particular religious belief and therefore unable to meditate in accordance with the requirements of the course. Potential participants were also screened by evaluating whether they met the aforementioned inclusion criteria and exclusion criteria after completing the demographic questionnaire and screening scales, concluding PHQ-9 and GAD-7, followed by written informed consent, as stipulated by the Declaration of Helsinki (2008).

From a total of 73 volunteers, a convenience sample consisting of 70 subjects met the inclusion criteria. Baseline written questionnaires were completed in a meeting room before a baseline EEG was recorded according to relatively free choice by students within the first stipulated week, considering the intensive curriculum of the students and the schedule of the experienced and certified mindfulness instructor. The 36 students who completing baseline EEG recording in the first week were allocated to a free 5-day course of mindfulness training group (MTG) and the remaining 34 students who completing baseline EEG recording in the follow given week were assigned to a waiting list group (WLG), according to convenience sampling.

**Instruction for post-intervention EEG paradigms**

For the baseline EEG paradigms, participants performed four tasks (total time: ~40min). The first two tasks (every task lasted 5min) were the resting state with eyes open (ORpre) and eyes closed (CRpre) in order: Participants were instructed to try to avoid body movement during the two tasks and not to fall asleep when doing the CRpre. The third task (~15min) was Go/Nogo task and the last task (~15min) was a modified 2-choice oddball task for smoking cue reactivity initially.

For the post-intervention EEG paradigms, participants performed thirteen tasks (total time: ~95min). The first four tasks (ORpost, CRpost, Go/Nogo, and a modified 2-choice oddball task) were performed using the same baseline EEG paradigms. The fifth and sixth task (every task lasted ~5min) were mindfulness practice with eyes open (OMpost) and eyes closed (CMpost) separately via giving the same instruction “Now practice mindfulness, which means focusing on your thoughts, feelings and body sensations in the present moment in a nonjudgmental way, without reacting to them.” (Hudak et al., 2021) after the slightly different pre-instruction: “Hello, welcome to our experiment. In the following experiment, you will see a 30-second instruction. When the time is up, it will switch to a "+" screen lasting 4.5 minutes. Please read the instruction carefully and then keep your eyes open (during OMpost) or closed (during CMpost) on the "+" screen. You could apply the mindfulness techniques that you've learned or used to do the following experiment. Press the space bar to start when you're ready.”. The seventh task (~4min) was body scanning: Participants were talked to listen carefully to the audio of body scanning and act on what they heard after pressing play to start when they were ready. The eighth task (~3min) was watching a video of themselves (refer to MTG) doing mindfulness training, with music playing in the background (The WLG watched the above video with blurred faces), which drew forth loving-kindness meditation as a ninth task (~17min): Participants were instructed to pay attention to listening to the audio of loving-kindness meditation, and act on what they heard, with eyes closed after pressing play to start, when they were ready. Finally, the additional mindfulness practice and resting state tasks (OMpostR, CMpostR, ORpostR, and CRpostR) have respectively been followed through.

**EEG preprocessing and the calculation of the three EEG measures of brain activities**

We employed EEGlab13.0.0b ([*https://sccn.ucsd.edu/eeglab*](https://sccn.ucsd.edu/eeglab)) and custom MATLAB R2013b (The MathWorks, Natick, MA, United States) scripts to preprocess the EEG data offline. First, recorded EEG data were filtered at a range of 1-100 Hz followed by a notch filter at 50 Hz, which was aimed to remove any low-frequency baseline shifts and electronic noise. Second, the data were re-sampled to 512Hz and epochs of 2 s were created for each subject. Third, we discarded epochs contaminated by eye blinks, horizontal eye movements, vertical eye movements, excessive muscle activity, or drifts using visual inspection. Fourth, independent component analysis (ICA) was performed on the dataset. The resulting ICs were analyzed for artifacts and contaminated ICs were subtracted from the dataset via the ADJUST plug-in ([*https://www.nitrc.org/projects/adjust/*](https://www.nitrc.org/projects/adjust/)). And then, the brain activity more than ±100 μV deviation from 0 was discarded using threshold rejection. Lastly, the data was re-references to the Cz.

And then, the processed data (MTG=18 and WLG=15) was converted into frequency domain representations using the Darbeliai v2019.02.01.1 plug-in ([*https://github.com/embar-/eeglab_darbeliai/wiki/0.%20EN*](https://github.com/embar-/eeglab_darbeliai/wiki/0.%20EN)) in EEGlab. On the “Task” part of Darbeliai interface: the “Frequency interval (Hz)” was auto; the “FFT window length (s)” was 1; the “Spectrum steps in 1 Hz” was 10; the “Channels” was selected according to which channels the study was analyzed and the “Spectrum band” was set in the frequency ranges of 1-4Hz, 4-8Hz, 8-13Hz, 13-20Hz, 20-30Hz, and 30-48Hz, representing delta, theta, alpha, low-beta, high-beta, and low-gamma bands, respectively. Finally, average EEG band powers, including Abs, Rel, and PSD, were calculated.

**The Results of EEG Power and Spectrum**

Alpha power over frontal region (Fig. 2A). For Abs/lg, between-group analysis showed that the alpha Abs/lg of MTG significantly decreased than WLG on CMpost at Fz (p = 0.019, d = 0.84), F3 (p = 0.032, d = 0.77), and F4 (p = 0.045, d = 0.71) sites, while within-group analysis showed that the alpha Abs/lg in the MTG group increased significantly on CMpostR compared with CRpost at all sites after the day's audio and video guidance (Fp1: p = 0.006, d = 1.03; Fp2: p = 0.004, d = 1.09; F3: p = 0.012, d = 0.91; F4: p = 0.016, d = 0.87; Fz: p = 0.006, d = 1.04). Besides, it decreased on CMpost compared with CRpre at both Fp1 (p=0.004, d = 1.10) and Fp2 (p=0.009, d = 0.96), while there was no difference in WLG. For Rel, between-group analysis showed that there was significant difference between MTG and WLG on the alpha Rel over Fp1 (pCRpost = 0.032, d = 0.78), Fp2 (pCRpost = 0.035, d = 0.76), and F3 (pCRpost = 0.033, d = 0.76) but not over other sites. Within-group analysis showed that the alpha Rel of MTG in the frontal region increased significantly on CMpostR compared with CRpost (Fz: p = 0.014, d = 0.90; Fp1: p = 0.012, d = 0.91; Fp2: p = 0.011, d = 0.93; F3: p = 0.007, d = 0.99; F4: p = 0.007, d = 1.00) after the day's audio and video guidance, besides, it also increased on CMpostR compared with CRpost (Fz: p = 0.016, d = 0.88; Fp1: p = 0.006, d = 1.44; Fp2: p = 0.012, d = 0.92; F3: p = 0.003, d = 1.13), while there was no difference in WLG.

Theta power over midline region (Fig. 2B). For Abs/lg, between-group analysis showed that there was no statistically significant difference between two groups in theta band over midline region. Within-group analysis showed that theta Abs/lg at the Pz, CRpostR (p=0.001, d = 1.49) and CMpostR (p=0.004, d = 1.23) were both higher than CRpre, and at Oz, CRpostR was higher than CRpre (p=0.010, d = 1.06) in WLG, while there was no significant difference at all channels of MTG. For Rel, between-group analysis showed that no significant difference was observed between MTG and WLG in the theta band of all sites. Within-group analysis showed that there was no significant difference in both MTG and WLG on relative theta band power value of all sites except Pz or/and Oz, which exhibited that in Pz the theta Rel of CMpost increased than CRpre in MTG (p = 0.005, d = 1.04), and in Oz the theta Rel of CMpost increased than CRpost in MTG (p = 0.009, d = 0.97). Low-beta power over midline region (Fig. 2C). Between-group analysis showed that the low-beta Abs/lg of MTG was significantly lower than WLG at Fz, Pz, and Oz sites on CRpost (Fz: p = 0.038, d = 0.74; Pz: p = 0.031, d = 0.77; Oz: p = 0.048, d = 0.70), CMpost (Fz: p = 0.014, d = 0.89; Pz: p = 0.018, d = 0.85; Oz: p = 0.013, d = 0.90), and CRpostR (Fz: p = 0.023, d = 0.82; Pz: p = 0.049, d = 0.70; Oz: p = 0.008, d = 0.97).

Low-beta power over midline region (Fig. 2C). Between -group analysis showed that the low-beta Abs/lg of MTG was significantly lower than WLG at Fz, Pz, and Oz sites on CRpost (Fz: p = 0.038, d = 0.74; Pz: p = 0.031, d = 0.77; Oz: p = 0.048, d = 0.70), CMpost (Fz: p = 0.014, d = 0.89; Pz: p = 0.018, d = 0.85; Oz: p = 0.013, d = 0.90), and CRpostR (Fz: p = 0.023, d = 0.82; Pz: p = 0.049, d = 0.70; Oz: p = 0.008, d = 0.97), while for low-beta Rel, there was no significant difference between MTG and WLG at all sites except Pz (pCRpre = 0.010, d = 0.94, pCRpost = 0.017, d = 0.86) and Oz (pCRpre = 0.031, d = 0.77, pCMpostR = 0.045, d = 0.73, pCRpostR = 0.030, d = 0.77). However, within-group analysis showed that there was no significant difference in both MTG and WLG on both low-beta Abs and Rel of all sites.
