# Supplementary material for: Changes in Mental Health and EEG Biomarkers of Undergraduates Under Different Patterns of Mindfulness
Source: Brain Topogr. 2023 Dec 25;37(1):75–87. doi: 10.1007/s10548-023-01026-y (PMC10771601; doi:10.1007/s10548-023-01026-y)
Supplement: Supplementary file 3 — Supplementary file3 (PDF 864 KB)—Study flowchart. MTG, mindfulness training group; WLG, waiting list group; ICs: independent components. [file 10548_2023_1026_MOESM3_ESM.pdf]

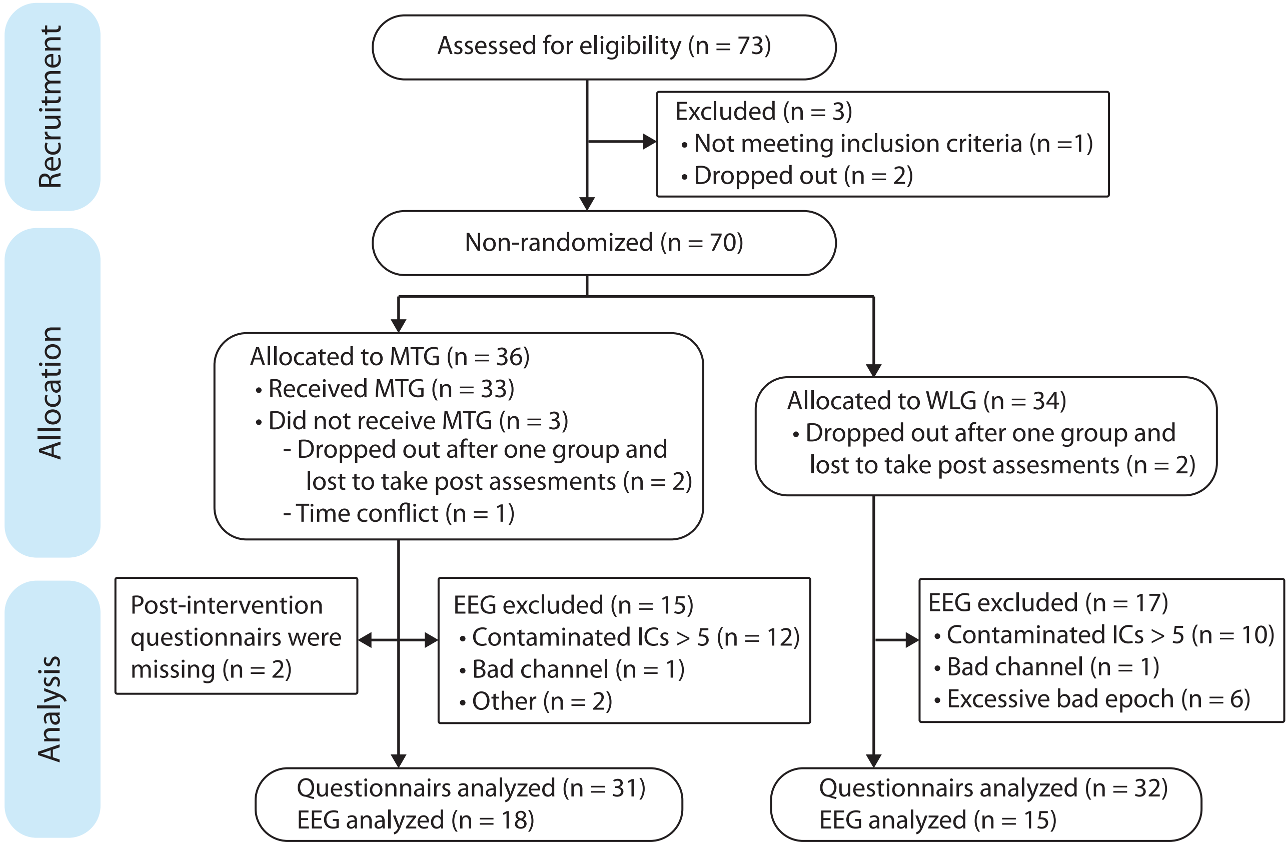
Recruitment
Allocation
Analysis
Assessed for eligibility (n = 73)
Excluded (n = 3)
• Not meeting inclusion criteria (n =1)
• Dropped out (n = 2)
Non-randomized (n = 70)
Allocated to MTG (n = 36)
• Received MTG (n = 33)
• Did not receive MTG (n = 3)
- Dropped out after one group and lost to take post assesments (n = 2)
- Time conflict (n = 1)
Allocated to WLG (n = 34)
• Dropped out after one group and lost to take post assesments (n = 2)
Post-intervention questionnairs were missing (n = 2)
EEG excluded (n = 15)
• Contaminated ICs > 5 (n = 12)
• Bad channel (n = 1)
• Other (n = 2)
EEG excluded (n = 17)
• Contaminated ICs > 5 (n = 10)
• Bad channel (n = 1)
• Excessive bad epoch (n = 6)
Questionnairs analyzed (n = 31)
EEG analyzed (n = 18)
Questionnairs analyzed (n = 32)
EEG analyzed (n = 15)
