# Supplementary figures and images for: Changes in Mental Health and EEG Biomarkers of Undergraduates Under Different Patterns of Mindfulness
Source: Brain Topogr. 2023 Dec 25;37(1):75–87. doi: 10.1007/s10548-023-01026-y (PMC10771601; doi:10.1007/s10548-023-01026-y)

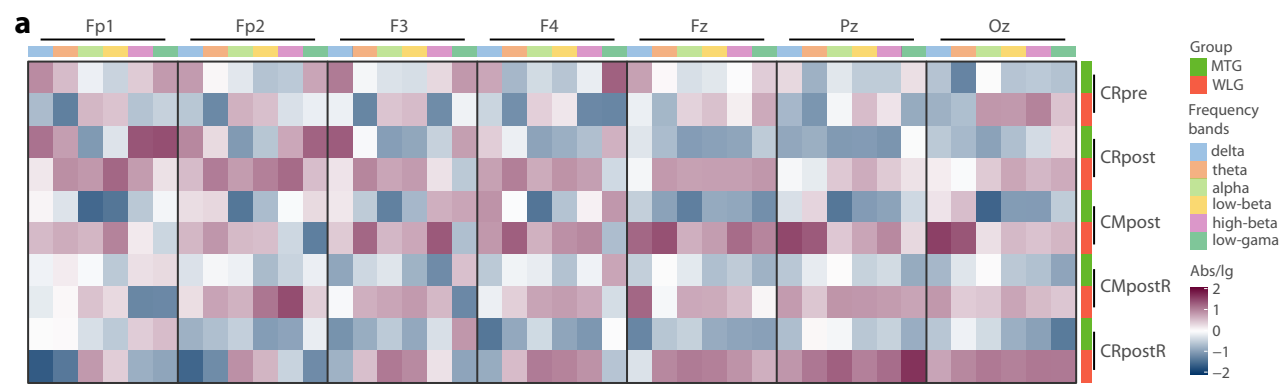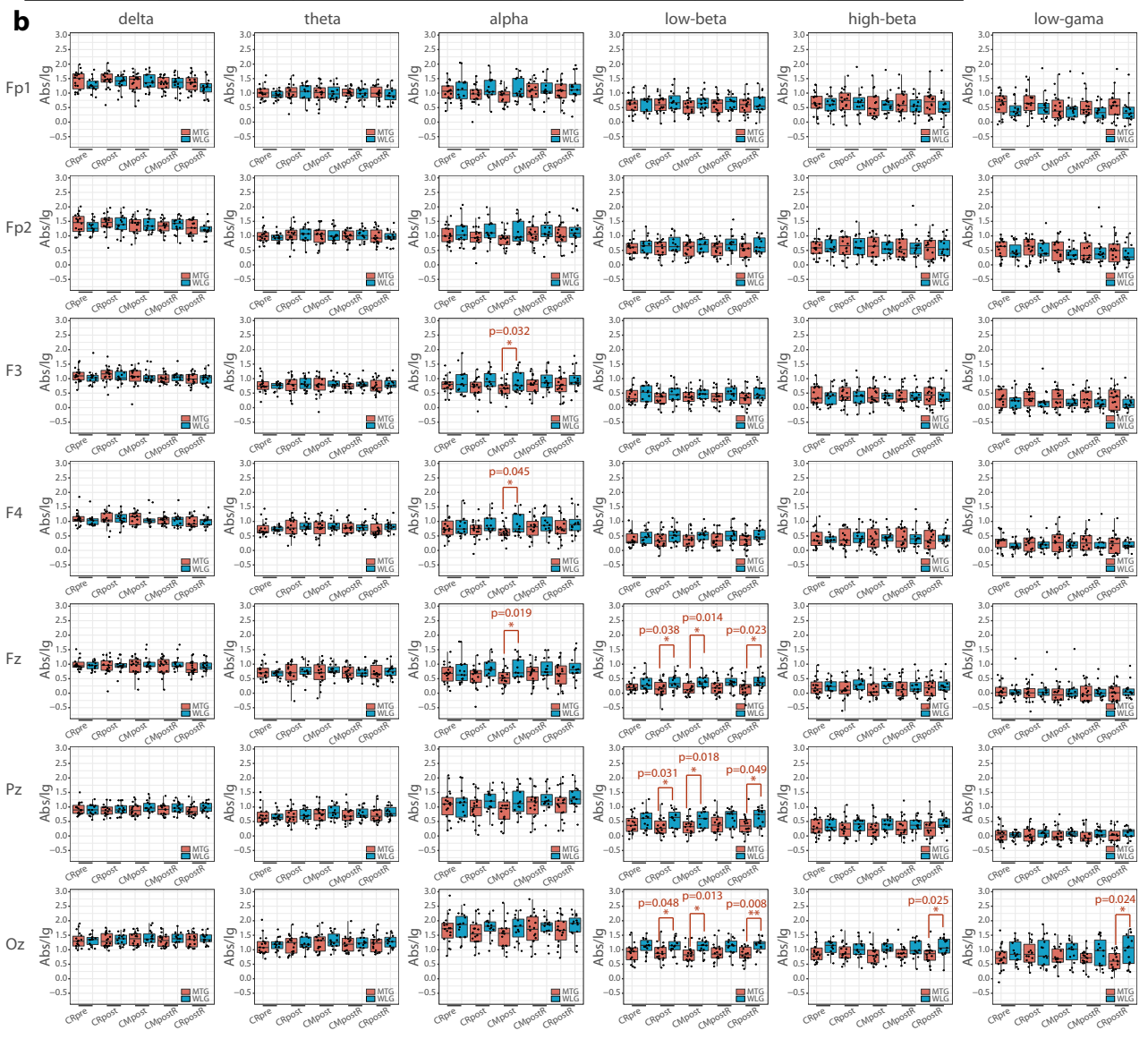

Supplement: Supplementary file 4 — Supplementary file4 (PDF 1658 KB)—The heatmap and box-plots of log10-transformed absolute power for between-group analysis results. (a) Heatmap of absolute power of five frequency bands in sites of frontal and midline regions. Abs/lg bar: values are depicted as standardized Z-scores for each power, where blue represents low value and purple represents high value. (b) Box-plots of absolute power between groups (two-sided unpaired t-tests; *p < 0.05, **p < 0.01, ***p < 0.001). Note: MTG, mindfulness training group; WLG, waiting list group; the resting state with eyes closed (CR); autonomic mindfulness practice with eyes closed (CM); five frequency bands: delta, theta, alpha, low-beta, high-beta, and low-gamma bands; frontal region: Fp1, Fp2, F3, Fz, and F4; midline region: Fz, Pz, and Oz; Fz is the frontal midline electrode; EEG tasks: CRpre, CRpost, CMpost, CMpostR, and CRpostR. Abs/lg, means log10-transformed absolute power. [file 10548_2023_1026_MOESM4_ESM.pdf]

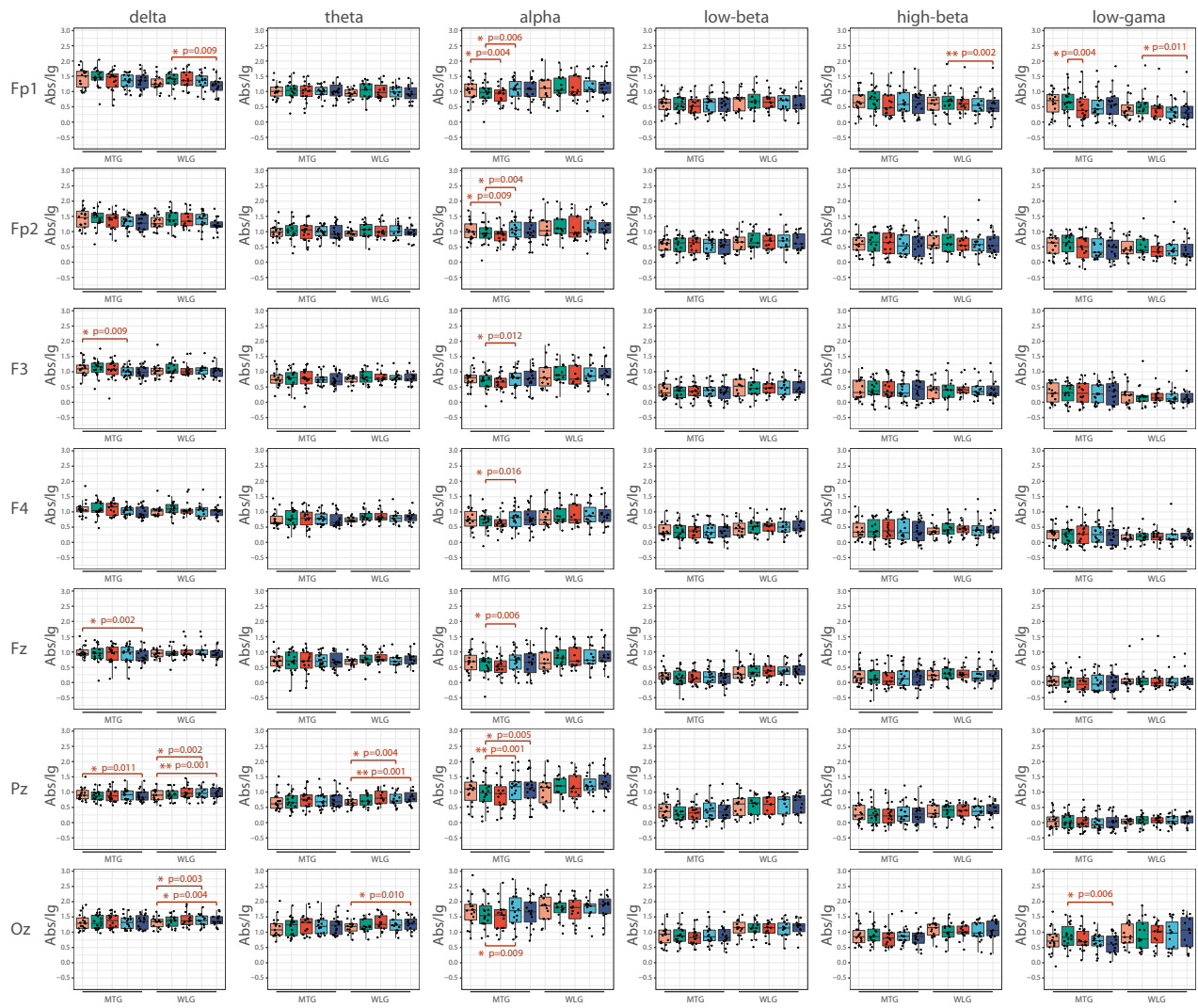

Supplement: Supplementary file 5 — Supplementary file5 (PDF 1242 KB)—The box-plots of log10-transformed absolute power for within-group analysis results. Data were inspected for multiple paired t-test (four pairwise comparisons based on CRpre: *p < 1.25×10^-2^, **p < 2.5×10^-3^, ***p < 2.5×10^-4^; three pairwise comparisons based on CRpost: *p < 1.67×10^-2^, **p < 3.3×10^-3^, ***p < 3×10^-4^, both following Bonferroni correction). See Figure S2 Note for the acronym. [file 10548_2023_1026_MOESM5_ESM.pdf]

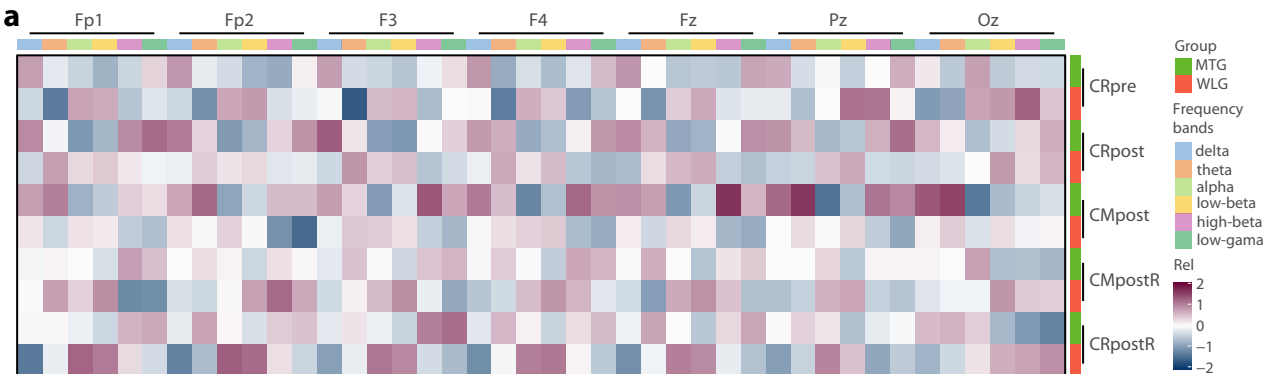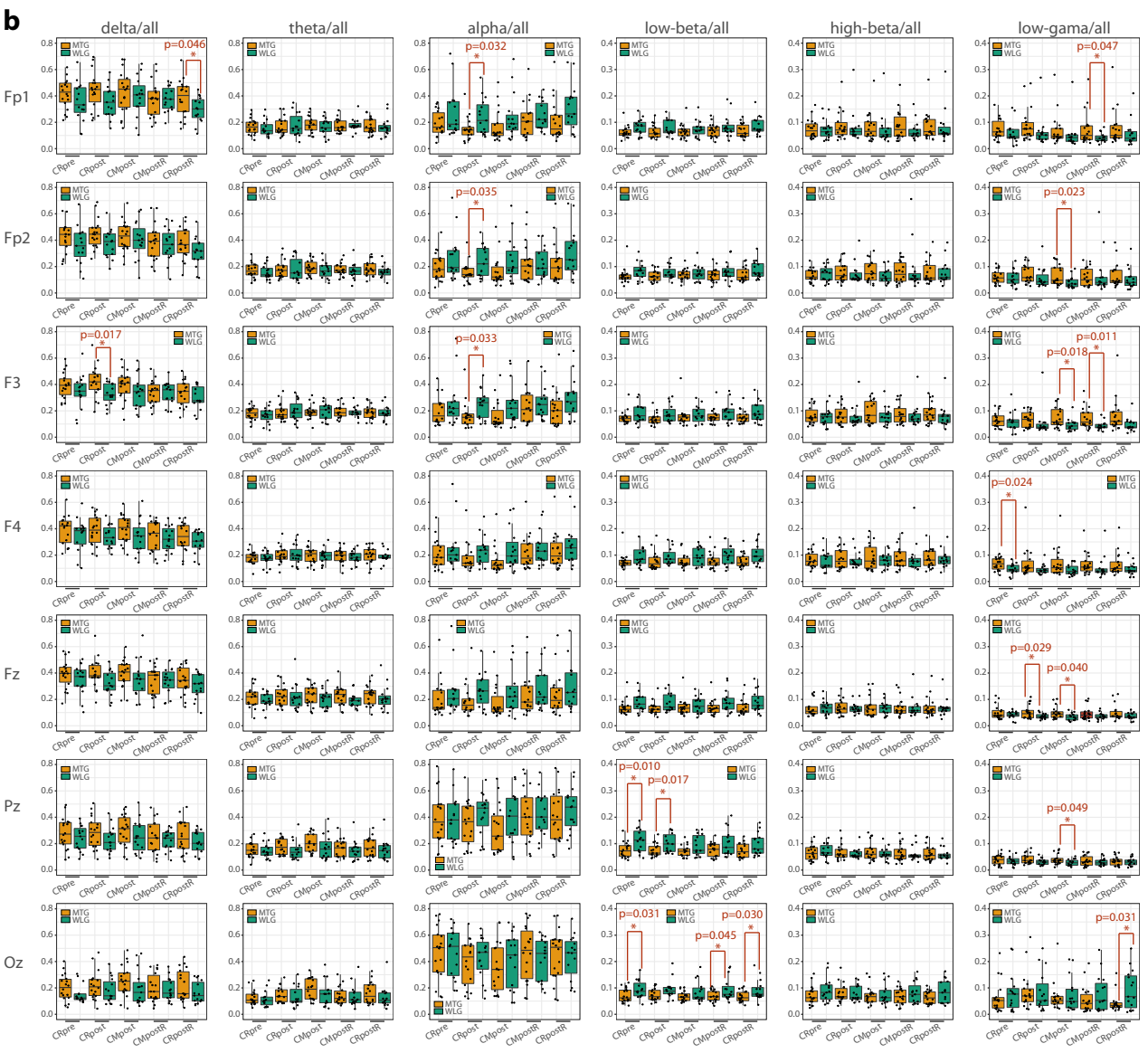

Supplement: Supplementary file 6 — Supplementary file6 (PDF 1454 KB)—The boxplots of relative power of five frequency bands in sites of frontal and midline regions between groups.(a) Heatmap of relative power of five frequency bands in sites of frontal and midline regions. Rel bar: values are depicted as standardized Z-scores for each power, where blue represents low value and purple represents high value. (b) Box-plots of relative power between groups (two-sided unpaired t-tests; *p < 0.05, **p < 0.01, ***p < 0.001). Rel, relative power; delta/all, theta/all, alpha/all, low-beta/all, high-beta/all, and low-gamma/all, representing delta, theta, alpha, low-beta, high-beta, and low-gamma bands’ relative power, respectively; see Figure S2 Note for the acronym. [file 10548_2023_1026_MOESM6_ESM.pdf]

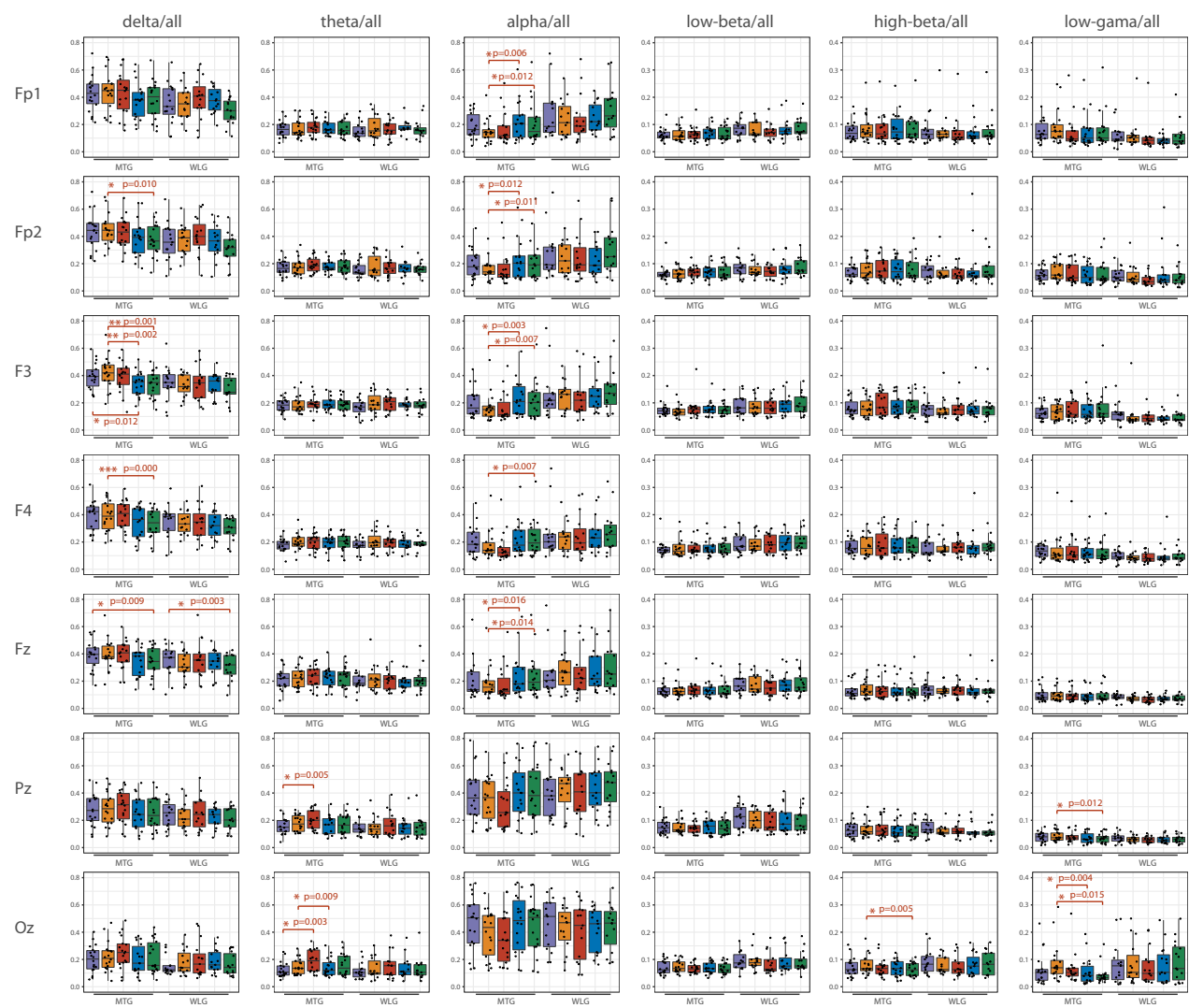

Supplement: Supplementary file 7 — Supplementary file7 (PDF 1180 KB)—The box-plots of the relative power for within-group analysis results.Data were inspected for multiple paired t-test (four pairwise comparisons based on CRpre: *p < 1.25×10^-2^, **p < 2.5×10^-3^, ***p < 2.5×10^-4^; three pairwise comparisons based on CRpost: *p < 1.67×10^-2^, **p < 3.3×10^-3^, ***p < 3×10^-4^, both following Bonferroni correction). See Figure S2 Note for the acronym. [file 10548_2023_1026_MOESM7_ESM.pdf]

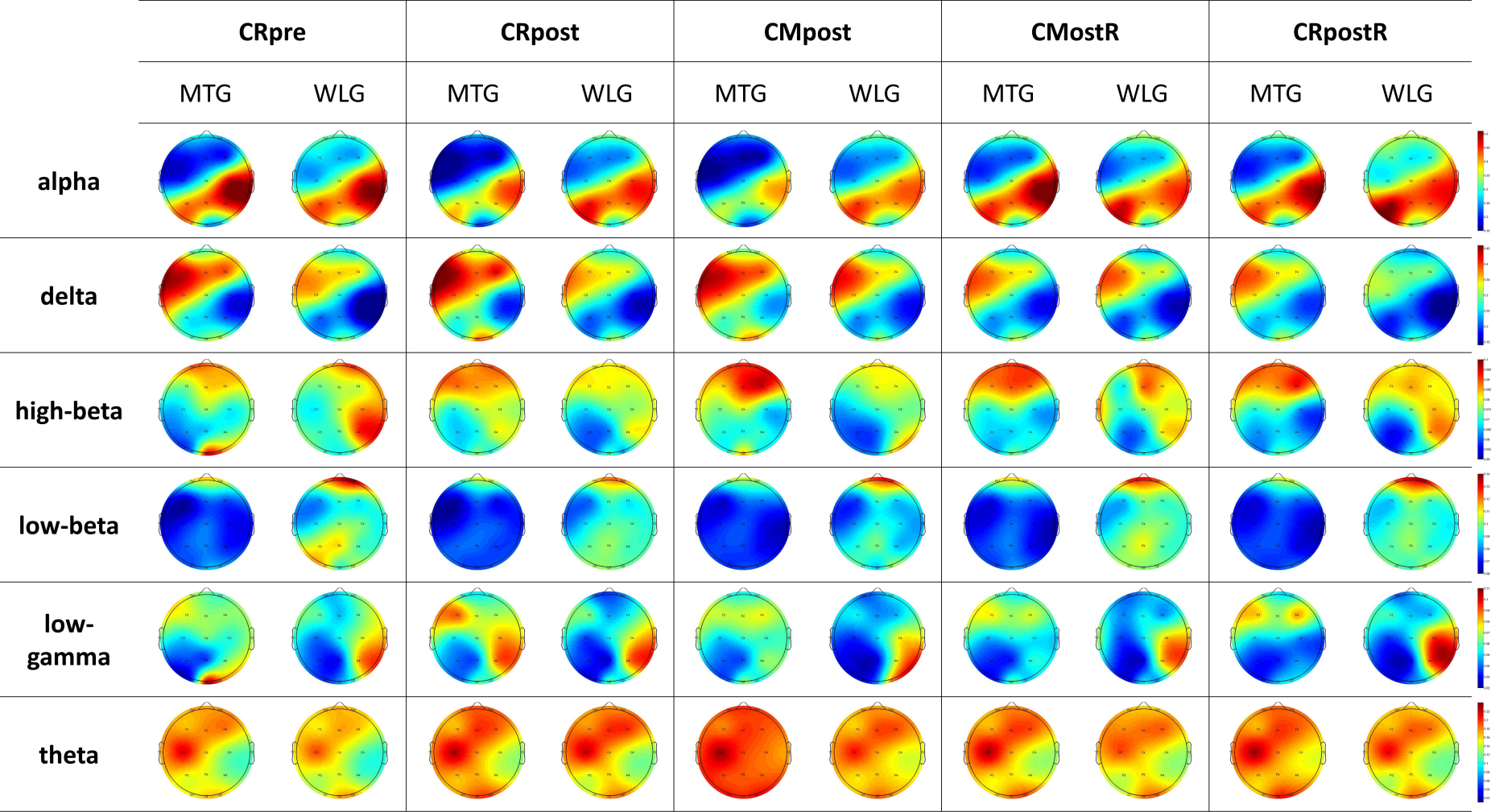

Supplement: Supplementary file 8 — Supplementary file8 (PDF 2330 KB)—Topographic maps showing scalp recorded relative power of five frequency bands for five EEG tasks under the eyes closed state. Scales: [0.15-0.51], [0.14-0.46], [0.05-0.10], [0.06-0.14], [0.02-0.11], [0.02-0.24]. See Figure S2 Note for the acronym. [file 10548_2023_1026_MOESM8_ESM.pdf]
