# Supplementary material for: Changes in Mental Health and EEG Biomarkers of Undergraduates Under Different Patterns of Mindfulness
Source: Brain Topogr. 2023 Dec 25;37(1):75–87. doi: 10.1007/s10548-023-01026-y (PMC10771601; doi:10.1007/s10548-023-01026-y)
Supplement: Supplementary file 9 — Supplementary file9 (PDF 1236 KB)—The line charts of the power spectral density for between- and within-group analysis results. (a) The PSD of five frequency bands in sites of frontal and midline regions within groups. Data were inspected for multiple paired t-test (pairwise comparisons based on CRpre: *p < 1.25×10^-2^, **p < 2.5×10^-3^, ***p < 2.5×10^-4^; and CRpost: *p < 1.67×10^-2^, **p < 3.3×10^-3^, ***p < 3×10^-4^, both following Bonferroni correction). (b) The PSD between groups (two-sided unpaired t-tests; *p < 0.05, **p < 0.01, ***p < 0.001). frequency bands: 1-4Hz, 4-8Hz, 8-13Hz, 13-20Hz, 20-30Hz, and 30-48Hz, representing delta, theta, alpha, low-beta, high-beta, and low-gamma bands, respectively; See Figure S2 Note for the acronym. [file 10548_2023_1026_MOESM9_ESM.pdf]

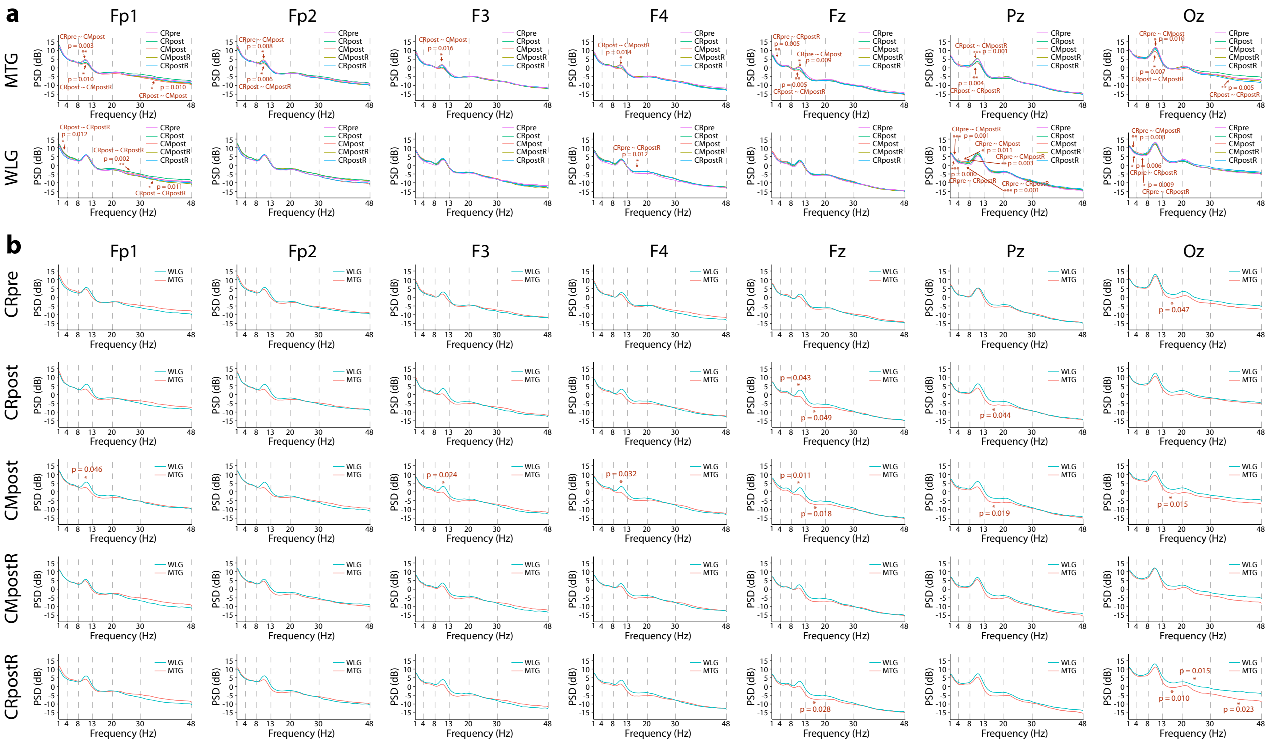

a
Fp1
Fp2
F3
F4
Fz
Pz
Oz
MTG
WLG
CRpre
CRpost
CMpost
CMpostR
CRpostR
PSD (dB)
Frequency (Hz)
CRpre ~ CMpost
p = 0.003
p = 0.010
CRpost ~ CMpostR
p = 0.010
CRpost ~ CMpost
CRpre ~ CMpost
p = 0.008
p = 0.006
CRpost ~ CMpostR
CRpost ~ CMpost
p = 0.016
CRpost ~ CMpostR
p = 0.014
CRpre ~ CRpostR
p = 0.005
CRpre ~ CMpost
p = 0.009
p = 0.005
CRpost ~ CMpostR
CRpost ~ CMpostR
p = 0.001
p = 0.004
CRpost ~ CRpostR
CRpre ~ CMpost
p = 0.010
p = 0.007
CRpost ~ CMpostR
p = 0.005
CRpost ~ CRpostR
CRpost ~ CRpostR
p = 0.012
CRpost ~ CRpostR
p = 0.002
p = 0.011
CRpost ~ CRpostR
CRpre ~ CRpostR
p = 0.012
CRpre ~ CMpostR
p = 0.001
CRpre ~ CMpost
p = 0.011
CRpre ~ CMpostR
p = 0.003
p = 0.000
CRpre ~ CRpostR
CRpre ~ CRpostR
p = 0.001
CRpre ~ CMpostR
p = 0.003
p = 0.006
CRpre ~ CRpostR
p = 0.009
CRpre ~ CRpostR
b
Fp1
Fp2
F3
F4
Fz
Pz
Oz
CRpre
CRpost
CMpost
CMpostR
CRpostR
WLG
MTG
p = 0.047
p = 0.043
p = 0.049
p = 0.044
p = 0.046
p = 0.024
p = 0.032
p = 0.011
p = 0.018
p = 0.019
p = 0.015
p = 0.028
p = 0.015
p = 0.010
p = 0.023
